# Consequences of herbal mixture supplementation on milk performance, ruminal fermentation, and bacterial diversity in water buffaloes

**DOI:** 10.7717/peerj.11241

**Published:** 2021-05-14

**Authors:** Faizul Hassan, Zhenhua Tang, Hossam M. Ebeid, Mengwei Li, Kaiping Peng, Xin Liang, Chengjian Yang

**Affiliations:** 1 Key Laboratory of Buffalo Genetics, Breeding and Reproduction Technology, Ministry of Agriculture and Guangxi Buffalo Research Institute, Chinese Academy of Agricultural Sciences, Nanning, Gunagxi, China; 2 Institute of Animal and Dairy Sciences, Univeresity of Agriculture, Faisalabad, Faisalabad, Pakistan; 3 Dairy Science Department, National Research Centre, Giza, Egypt

**Keywords:** Herbal mixture, Rumen bacteria, Fermentation, Milk yield, Milk fatty acids, Buffalo, High-throughput sequencing

## Abstract

This study was aimed to evaluate the potential of a herbal mixture (HM) to improve production performance, rumen fermentation, and milk fatty acid profile in water buffaloes. Sixteen Murrah buffaloes (in four groups) were fed for 10 weeks with the same basal diet supplemented with 0 (control); 20 (HM20), 30 (HM30), and 40 (HM40) g/buffalo per day. The herbal mixture contained an equal quantity of black pepper (fruit), ginger (tubers), cinnamon (bark), peppermint (leaves), ajwain (seeds) and garlic (bulbs). After two weeks of adaptation, daily milk yield, and weekly milk composition were recorded. On the last day of the experiment, rumen contents were collected to determine rumen fermentation parameters and bacterial diversity through 16S rRNA sequencing. Results revealed no effect of treatment on dry matter intake (DMI), rumen fermentation parameters, and daily milk yield. However, milk fat (%) showed a tendency to increase (*p* = 0.07) in HM20 as compared with the control group. A significant increase in mono and polyunsaturated fatty acids (C14:1, C16:1, C18:2n6 and C18:3) whereas a decrease in saturated fatty acids (C18:0) in milk was observed in HM20 as compared with the control group. No significant change in bacterial diversity parameters (alpha and beta diversity) was observed in response to the treatment. Despite the substantial variation observed in the relative abundance of bacteria among treatment groups, no significant effect of treatment was observed when compared with the control group. Correlation analysis revealed several positive and negative correlations of rumen bacteria with rumen volatile fatty acids (VFA) and milk yield traits. Bacterial genera including *Succinivibrionaceae, Butyrivibrio, Pseudobutyrivibrio*, and *Lachnospiraceae* showed a positive correlation with VFA and milk yield traits. Overall, we observed 52 positive and 10 negative correlations of rumen bacteria with milk fatty acid contents. Our study revealed the potential of the herbal mixture at a lower supplemental level (20 g/day) to increase milk fat (%) and unsaturated fatty acid content in buffalo.

## Introduction

Dietary supplementation of phytochemicals from different herbal plants has shown desirable effects on rumen fermentation, leading to increased milk yield and better health in dairy cattle (Calsamiglia et al., 2007; Oh et al., 2013; Patra & Yu, 2012; Hassan et al., 2020). Most of the studies (both in vitro and in vivo) have used single herbs for dietary supplementation while only few have used a combination of different herbs or their extracts. Moreover, to the best of our knowledge, no study is available on the effect of a combination of herbs on milk yield and composition, especially fatty acid profile. We hypothesized that using a combination of six medicinal plants with biological activities can modulate rumen bacteria to improve milk yield and composition of the buffalo. These herbs were selected based on their proven biological activities and their potential individual effects on animals already reported (Oh et al., 2013). Moreover, individual effects of these herbs or their extracts have been reported earlier in ruminants mainly on in vitro rumen fermentation characteristics. For example, feeding 200g/day of peppermint (*Mentha piperita L*.) in cattle decreased the ruminal ammonia nitrogen (NH_3_-N) concentration (Ando et al., 2003). Similarly, supplementation of ginger and garlic improved in vitro rumen fermentation characteristics by reducing NH_3_-N, methane and acetate to propionate ratio along with increasing fibrolytic bacteria and decreasing protozoa (Kim et al., 2012; Soroor & Moeini, 2015). Cinnamon and cumin powder and their essential oils have shown to decrease in vitro ruminal gas, NH_3_-N concentration and methane production (Danesh Mesgaran et al., 2009; Jahani-Azizabadi et al., 2011). Similarly, garlic has shown to inhibit deamination and decrease methanogenesis while cinnamon inhibited peptidolysis during in vitro ruminal fermentation (Busquet et al., 2005; Cardozo et al., 2004).

It is well established that the efficacy of individual active compound is lower than whole plant and/or its extract, mainly due to the synergistic effect of its individual compounds in combination (Busquet et al., 2005; O’Gara, Hill & Maslin, 2000; Ross et al., 2001). Therefore, to explore an overall sustainable effect of mixed phytochemical compounds, we envisaged using a mixture of selected medicinal plants. No study is available regarding the effect of these herbs on relative abundance and diversity of rumen bacteria and associated changes in rumen fermentation, milk yield, and fatty acid profile. Therefore, the present study was conducted to evaluate the effect of the herb mixture on rumen bacteria and their correlation with rumen fermentation and milk yield traits of lactating buffaloes.

## Materials & Methods

### Ethics statement

The animal study was reviewed and approved by the Ethics committee of the Chinese Academy of Agriculture Sciences, Guangxi Buffalo Research Institute, China (Approval Number BRI-2017006). All experimental procedure used in this experiment was strictly abide by the guidelines of Ethics Committee of the Chinese Academy of Agriculture Sciences, Guangxi Buffalo Research Institute, China.

### Animals, diets and experimental design

This research was carried out at Guangxi Buffalo Research Institute, Nanning, China (latitude 28°.48′N, longitude 108°.22′E). Sixteen Murrah buffaloes of almost similar body weight (560 ± 20 kg), parity (3–4), and stage of lactation (90–120 days) were randomly enrolled for this study under a randomized complete block design. The effect of four levels of herb mixture (HM) on rumen fermentation, rumen bacteriome, and milk production of Murrah buffaloes was investigated. We allocated four treatments to 16 buffaloes using the complete randomized design because all animals had almost similar average body weight. These four treatment groups of buffaloes (four buffaloes per group) fed with different doses of herb mixture included; HM20 (20 g/d/head), HM30 (30 g/d/head), HM40 (40 g/d/head) and control group (0 g/d/head). The same experimental diet consisting of maize silage, brewer’s grain, and concentrate mixture were fed to all animals for 10 weeks. Details of the chemical composition of the experimental diet were given in Table 1. All animals were managed under similar housing and management conditions. All animals had free access to water. Total mix ration was fed twice daily in the morning and evening before milking for ad libitum intake. During the 10 weeks of data collection, milk samples were collected weekly for the determination of milk composition. Each buffalo was milked twice with the milking machine and daily milk yield was recorded for all groups throughout the experimental period. Individual feed intake was recorded by measuring feed and leftovers both in the morning and evening daily during the last week of the experiment.

### Formulation of herbal mixture

The mixture of herbs was prepared by using an equal quantity of six herbs with known antioxidant, and antimicrobial activities. Herbs selected for formulation included; black pepper (fruit), ginger (tubers), cinnamon (bark), peppermint (leaves), ajwain (seeds) and garlic (bulbs). These herbs were procured in dry from Verbena Nutraceuticals Inc. (Islamabad, Pakistan), powdered and mixed to form a uniform mixture. Total polyphenolic contents (measured as Gallic acid equivalent) were determined using the Folin-Ciocalteau’s phenol reagent as reported previously (Hosoda et al., 2005) and averaged about 13.6 mg/g of the mixture.

### Collection of rumen contents and processing

At the end of the trial, rumen contents were collected from buffaloes using a stainless-steel stomach tube. About 500 mL of rumen contents were collected before the morning feeding in sterilized plastic bottles. After collection, samples were immediately transferred to the lab for further analysis. Subsequently, the rumen contents were strained through two layers of cheesecloth and subsamples of rumen contents for determination of volatile fatty acids (VFA), ammonia nitrogen (NH3-N) and microbial crude protein (MCP) were stored at −20 °C. Subsamples for DNA extraction stored at −80 °C till further processing.

**Table 1:** Formulation and chemical composition of the experimental diet.

**Items**	**Contents**
Ingredient of basal diet (g/kg of DM)	
Corn Silage	196
Brewer’s grain	395
Concentrate Feed Mixture (CFM)^a^	409
Total	1000
Chemical composition of basal diet (g/kg of DM, unless otherwise stated)	
Dry Matter (g/kg as fed)	425
Organic Matter	814
Crude Protein (CP)	167
Non Detergent Fiber (NDF)	131
Acid DetergentFiber (ADF)	87
Gross energy (kcal/kg DM)	4.36

**Notes.**

a CFM: concentrate feed mixture (corn 17.83%; wheat bran 7.51%; Soybean meal 5.72%; Lime stone 0.5%; CaHPO_4_ 0.6% ; NaHCO3 0.8%; NaCl 0.7%; Premix1 0.34%). ^1^The additive premix provided the following per kg of CFM: VA 550 000 IU, VE 3000 IU, VD3 150 000IU, 4.0 g Fe (as ferrous sulfate), 1.3 g Cu (as copper sulfate), 3.0 g Mn (as manganese sulfate), 6.0 g Zn (as zinc sulfate), 80 mg Co(as cobalt sulfate).

### Determination of rumen fermentation parameters

After the collection of rumen contents, pH was measured immediately using a pH meter (HI 9024C; HANNA Instruments, Woonsocket, Rhode Island, USA). A subsample of rumen fluid (4 mL) was acidified with 4 ml of HCl (0.2 mol/L) and stored in a freezer (−20 °C) for determination of NH3-N using indophenols method (Weatherburn, 1967). Microbial protein content was analyzed with a spectrophotometer at 595 nm using 1 mg/mL bovine serum albumin solution (Sigma-Aldrich Co., LLC, St. Louis, Missouri, USA) as standard equivalent (Makkar et al., 1982). The concentrations of VFA (C2, C3, C4, C5, iC4, and iC5) were measured using a GC system (Agilent 7890A, Agilent Technologies, USA), as described by Qin, (1982).

### Milk yield and composition

Milk yield for morning and evening milking was recorded daily for each buffalo; however, milk samples for determination of milk composition were collected weekly. Milk composition (milk total solids, protein, fat and lactose) was analyzed for morning and evening milk samples separately using MilkoScanTM F120 (FOSS, Hillerød, Denmark). Milk samples of morning and evening were pooled (relative to the quantity of milk produced) for each week separately and stored at −20 °C until processed for the analysis of fatty acid profile. Briefly, 20 mL of buffalo milk was centrifuged in a 50 mL falcon tube at 17,800 × g for 30 min at 4 °C. After centrifugation, the above fat layer (1.0 g) was transferred to a 1.5 mL eppendorf tube and left at room temperature (∼20 °C) for approximately 20 min to allow fat to melt. After that, it was centrifuged at 19,300 × g for 20 min at room temperature in a microcentrifuge. Centrifugation of fat separated the sample into 3 layers: top layer containing lipid; middle layer containing protein, fat, and other water-insoluble solids; and bottom aqueous layer (Feng, Lock & Garnsworthy, 2004). Milk fatty acids were trans-esterified with sodium methoxide according to the method previously reported (Zahran & Tawfeuk, 2019). Briefly, 2.0 mL of n-hexane was added to 40 ul of butter fat and vortexed for 30 s followed by the addition of 2 mL of sodium methoxide (0.4 mol). After vortexing, the mixture was allowed to settle for 15 min. The upper phase, containing the fatty acid methyl ester (FAME), was recovered and analyzed by an Agilent 7890B Gas chromatography (GC-FID) with a polar capillary column SP®-2560 100 m, 0.25 mm id, 0.2 µm film thickness. Helium was used as a carrier gas at a flow rate of 20 cm sec-1 and split ratio 100:1. The column temperature profile was held at 100 °C for 5 min, ramp to 240 °C @ 4 °C min-1; hold at 240 °C for 30 min. A sample volume of 1.0 µL was injected. The FAME was identified by comparing their relative and absolute retention times with FAME standards (from C4:0 to C22:0). Fatty acid contents are presented as percentage of total fat weight (wt%/wt%).

### DNA extraction from rumen contents

The DNA was extracted from frozen samples of rumen contents including both liquid and solid phase. One ml of rumen contents was centrifuged at 12,000 × g for 5min to pellet the microbial cells by removing supernatant. These pelleted cells were treated with the CTAB method to extract DNA as reported previously (Yu & Morrison, 2004). Briefly, microbial cells were lysed by using zirconium bead beating in CTAB. After treatment with RNAse enzyme (10 mg/ml), impurities were removed by treating with Phenol:Chloroform:Isoamyl alcohol (25:24:1) at least three times. DNA was precipitated with isopropanol followed by washing with 70% ethanol to remove remaining salts etc. The quality of DNA was checked by the NanoDrop spectrophotometer (NanoDrop2000, Thermo Scientific, USA).

### High throughput sequencing of the 16S rRNA gene to analyze rumen bacterial diversity

IlluminaMiSeq sequencing was carried out after library preparation from purified DNA using barcoded primers for the V3–V4 region of 16S rRNA gene (Klindworth et al., 2013). DNA libraries were sequenced using a 2 × 300 paired-end sequencing module (Illumina, San Diego). After performing quality control, optimized sequence reads were aligned against the SILVA database, Release128 (http://www.arb-silva.de) for identification of Operational Taxonomic Units (OTU) using cluster identity threshold of 97% as reported previously (Quast et al., 2012; Yilmaz et al., 2013). The taxonomy of each sequence (OTU representative) was analyzed by RDP Classifier (http://rdp.cme.msu.edu/) against the database (confidence threshold of 0.7). All the above steps regarding the taxonomic assignment of rumen bacteria were performed with the bioinformatics pipeline of Qiime software (http://qiime.org/scripts/assign_taxonomy.html) as described previously (Caporaso et al., 2010). Bacterial diversity was determined in different treatment groups by analyzing alpha and beta diversity indices from the complete OTU table. Bacterial richness and evenness in each sample were analyzed by measuring Chao and abundance-based coverage estimator (ACE) while alpha diversity was estimated by determining Shannon and Simpson indices (Chao, 1984; Chao & Lee, 1992; Shannon, 1948; Simpson, 1949). Microbial evenness within each sample was assessed by Simpson and Shannon’s evenness (Pielou’s J) indices (Smith & Wilson, 1996). Beta diversity index was calculated to analyze rumen bacterial diversity across different samples using Bray-Curtis dissimilarities (Bray & Curtis, 1957). Bray-Curtis dissimilarities among different treatment groups were evaluated non-parametrically by utilizing permutation analysis of variance method (PERMANOVA using 999 permutations) as previously reported (Anderson, 2001).

### Statistical analysis

Effect of herb mixture on all parameters related to milk yield, dry matter intake (DMI), rumen fermentation, and bacterial alpha diversity, was analyzed using the PROC GLM procedure of SAS (SAS Institute Inc., Cary, NC, USA) having treatment as fixed effect and animal as a random effect nested in the treatment group. Firstly, we included the week as a factor in the model, but no significant effect of week was observed on any performance traits, so we excluded week from the final model. The Duncan’s multiple range test was used as a post hoc measure to detect the differences among treatment groups. We also analyzed three orthogonal contrasts including all treatments vs. control, linear effect of treatment dose, and quadratic effect of treatment dose for rumen fermentation and milk yield parameters. Treatment effects were declared significant at *p* < 0.05 and trends were discussed at 0.05 ≤ *p* < 0.1. The effect of dietary treatment on the abundances of bacterial order and genera was determined using the Kruskal-Wallis H test with false discovery rate (FDR) correction and Scheffe as a post-hoc test to elucidate differences across treatment groups. Spearman correlation coefficients (r) were measured with the vegan package of R software (Version 3.2) to analyze the association of relative abundance of bacterial genera with rumen fermentation and milk yield parameters. The correlation matrix was visualized using the pheatmap package of R software by displaying a heat map. In the two-dimensional heat map, change in defined color and its depth indicates the nature and strength of the correlation, respectively. Asterisk sign was used when the r value was greater than 0.1 and the *p* values were less than 0.05 (* 0.01 <*p* ≤ 0.05, ** 0.001 <*p* ≤ 0.01, *** *p* ≤ 0.001).

## Results

### Rumen fermentation

No effect (*p* > 0.05) of treatment was observed on any of the rumen fermentation parameters (Table 2).

**Table 2:** Effect of supplementation of herbal mixture on rumen fermentation parameters in lactating buffaloes.

**Parameter**	**Treatments**					***P* value**			
	**Control**	**HM20**	**HM30**	**HM40**	**SEM**	**Treat.**	**Linear**	**Quad.**	**Contrast**
pH	6.70	6.57	6.76	6.66	0.05	0.65	0.85	0.91	0.80
TVFAs (mmol/L)	34.52	37.67	33.53	38.06	1.49	0.70	0.66	0.83	0.60
Acetate (mmol/L)	16.60	17.90	16.23	17.93	0.63	0.76	0.71	0.89	0.63
Propionate (mmol/L)	10.07	11.62	9.50	11.60	0.57	0.51	0.65	0.82	0.54
Isobutyrate (mmol/L)	0.82	0.82	0.83	0.83	0.03	1.00	0.93	1.00	0.95
Butyrate (mmol/L)	5.45	5.57	5.33	5.96	0.31	0.93	0.68	0.72	0.82
Isovalerate (mmol/L)	1.00	1.05	1.00	1.03	0.06	0.99	0.94	0.96	0.87
Valerate (mmol/L)	0.60	0.70	0.63	0.66	0.05	0.91	0.79	0.77	0.59
Acetate/Propionate	1.65	1.57	1.73	1.53	0.04	0.53	0.66	0.53	0.74
MCP (mg/mL)	32.20	36.45	35.56	33.03	2.46	0.93	0.95	0.56	0.65
NH_3_-N (mg/mL)	19.57	19.18	18.02	18.48	1.23	0.98	0.74	0.88	0.75

**Notes.**

Values in the same row with different superscripts differ significantly (*p* < 0.05).

TVFA Total volatile fatty acids MCP Microbial crude protein NH_3_-N Ammonia nitrogen

(HM20 = herb mixture fed 20 g/d/head, HM30 = herb mixture fed 30 g/d/head, HM40 = herb mixture fed 40g/d/head, control = without herb mixture).

Treat. Treatment effect Linear Linear effet of treatment Quad. Quadratic effect of the treatment Contrast All treatments vs. control

### DMI, milk yield and composition

Results revealed no effect of herb mixture on the DMI, average milk yield and composition of buffaloes except milk fat (%) that tended to increase in HM20 (8.91%) as compared with HM30 (7.67%), HM40 (8.70%) and control (7.28%) group (*p* = 0.07, Table 3).

**Table 3:** Effect of herbal mixture on DMI and milk yield parameters of lactating buffaloes.

**Parameter**	**Control**	**HM20**	**HM30**	**HM40**	**SEM**	***P*Value**			
						Treat.	Linear	Quad.	Contrast
Dry matter intake (kg/d)	8.54	8.68	8.90	8.47	0.11	0.61	0.99	0.25	0.61
Milk yield (kg/d)	8.39	7.60	6.13	6.30	0.64	0.58	0.21	0.72	0.28
Fat corrected milk (kg/d)	12.42	13.05	9.59	10.66	0.97	0.61	0.35	0.91	0.58
Energy corrected milk (kg/d)	13.27	13.84	10.24	11.29	1.04	0.63	0.35	0.92	0.57
Protein (%)	4.49	4.98	4.57	4.88	0.13	0.55	0.54	0.75	0.33
Protein yield (kg/d)	0.38	0.38	0.29	0.31	0.03	0.69	0.33	0.91	0.51
Fat (%)	7.28	8.91	7.67	8.7	0.27	0.07	0.17	0.53	0.05
Fat yield (kg/d)	0.60	0.67	0.48	0.54	0.05	0.58	0.41	0.98	0.72
Total solids (%)	17.59	19.86	17.72	19.32	0.50	0.29	0.49	0.73	0.24
Solid not fat (%)	9.61	9.93	9.23	9.68	0.30	0.89	0.87	0.93	0.99
Lactose (%)	4.82	4.83	4.51	4.63	0.17	0.92	0.61	0.89	0.72

**Notes.**

Values in the same row with different superscripts differ significantly (*p* < 0.05).

Energy corrected milk (ECM) was calculated by using the following equation (Tyrrell & Reid, 1965); ECM = 0.327 × Milk yield (kg) + 12.95 × Fat yield (kg) + 7.20 × Protein (kg).

Similarly, 4% fat corrected milk (FCM) was calculated by following equation (NRC, 2001).

FCM (4%) = 0.4 × Milk yield + 15 × (Milk Fat/100) *x* Milk yield.

(HM20 = herb mixture fed 20 g/d/head, HM30 = herb mixture fed 30 g/d/head, HM40 = herb mixture 40 g/d/head, control = without herb mixture).

Treat. Treatment effect Linear Linear effet of treatment Quad. Quadratic effect of the treatment

### Milk fatty acid composition

In the present study, we aimed to quantify major 15 fatty acids including 9 saturated fatty acids (SFA), 3 monounsaturated fatty acids (MUFA), and 3 polyunsaturated fatty acids (PUFA) through GC analysis. Our study revealed C14:0, C16:0 and C18:0 as major SFA while C18:1 as the major unsaturated fatty acid (UFA) in milk of buffaloes (Table 4). Total saturated fatty acid (TSFA) contents ranged from 62 to 64% while UFA from 36 to 38%. No significant effect of treatment on total contents of short-chain fatty acids (SCFA), medium-chain fatty acids (MCFA) and long-chain fatty acids (LCFA) was observed as compared with the control group (*p* > 0.05). However, a decrease in major SFA, stearic acid (C18:0), was observed in HM20 and HM30 (*p* = 0.001), but no effect on other SFA was observed. A significant increase in mono and poly unsaturated fatty acids (C14:1, C16:1 and C18:2n6 and C18:3) in milk was observed in HM20 as compared with the control. However, the treatment showed no effect on n-3 to n-6 ratio in milk (*p* = 0.14).

**Table 4:** Fatty acids profile (g per 100 g FAME) of milk across different treatment groups.

**Fatty Acid**	**Common Name**	**Control**	**HM20**	**HM30**	**HM40**	**SEM**	***P*value**
**C4:0**	Butyric acid	0.83	0.86	0.88	0.89	0.03	0.38
**C6:0**	Caproic acid	0.96	0.95	0.93	0.98	0.02	0.81
**C8:0**	Caprylic acid	0.64	0.66	0.64	0.71	0.01	0.31
**C10:0**	Capric acid	1.41	1.47	1.45	1.61	0.03	0.24
**C12:0**	Lauric acid	2.01	2.20	2.16	2.36	0.05	0.19
**C14:0**	Myristic acid	10.15	10.59	10.26	10.32	0.15	0.71
**C14:1**	Myristoleic acid	1.05^c^	1.31^a^	1.23^ab^	1.14^bc^	0.02	0.001
**C16:0**	Palmitic acid	32.14	31.33	31.55	30.70	0.26	0.24
**C16:1**	Palmitoleic acid	1.97^b^	2.29^a^	2.20^ab^	1.194^b^	0.05	0.03
**C17:0**	Margaric acid	0.30	0.34	0.33	0.34	0.01	0.07
**C18:0**	Stearic acid	15.70^a^	14.05^b^	14.27^b^	16.35^a^	0.28	0.001
**C18:1**	Oleic acid	29.39	30.23	30.36	29.18	0.34	0.56
**C18:2n6**	Linoleic acid	1.41^b^	1.58^a^	1.50^ab^	1.51^ab^	0.02	0.04
**C18:3n3**	*α*-Linolenic acid	0.44	0.48	0.45	0.50	0.04	0.10
**C18:3**	Linolenic acid	1.60^ab^	1.72^a^	1.81^a^	1.48^b^	0.01	0.03
**Group of fatty acids, g/100 g of fatty acids**							
**Total SFA**		64.15	62.42	62.48	64.26	0.41	0.25
**Total UFA**		35.85	37.58	37.52	35.74	0.41	0.25
**SCFA**		3.78	3.90	3.90	4.18	0.08	0.38
**MCFA**		47.38	47.73	47.41	46.46	0.41	0.67
**LCFA**		48.83	48.39	48.69	49.37	0.47	0.85
**n-6/n-3**		0.28	0.29	0.26	0.43	0.03	0.14

**Notes.**

Values in the same row with different superscripts differ significantly (*p* < 0.05).

SFA Saturated fatty acids UFA unsaturated fatty acids SCFA short-chain fatty acids MCFA medium-chain fatty acids LCFA long-chain fatty acids

SCFA included the C4:0, C6:0, C8:0, and C10:0 fatty acids; MCFA included all linear fatty acids from C12:0 to C16:1; LCFA included all linear fatty acids from C17:0 to C18:3; (HM20 = herb mixture fed 20g/d/head, HM30 = herb mixture fed @ 30g/d/head, HM40 = herb mixture @ 40 g/d/head, control= without herb mixture).

### Rumen bacterial diversity

#### OTU statistics

**Figure 1:**
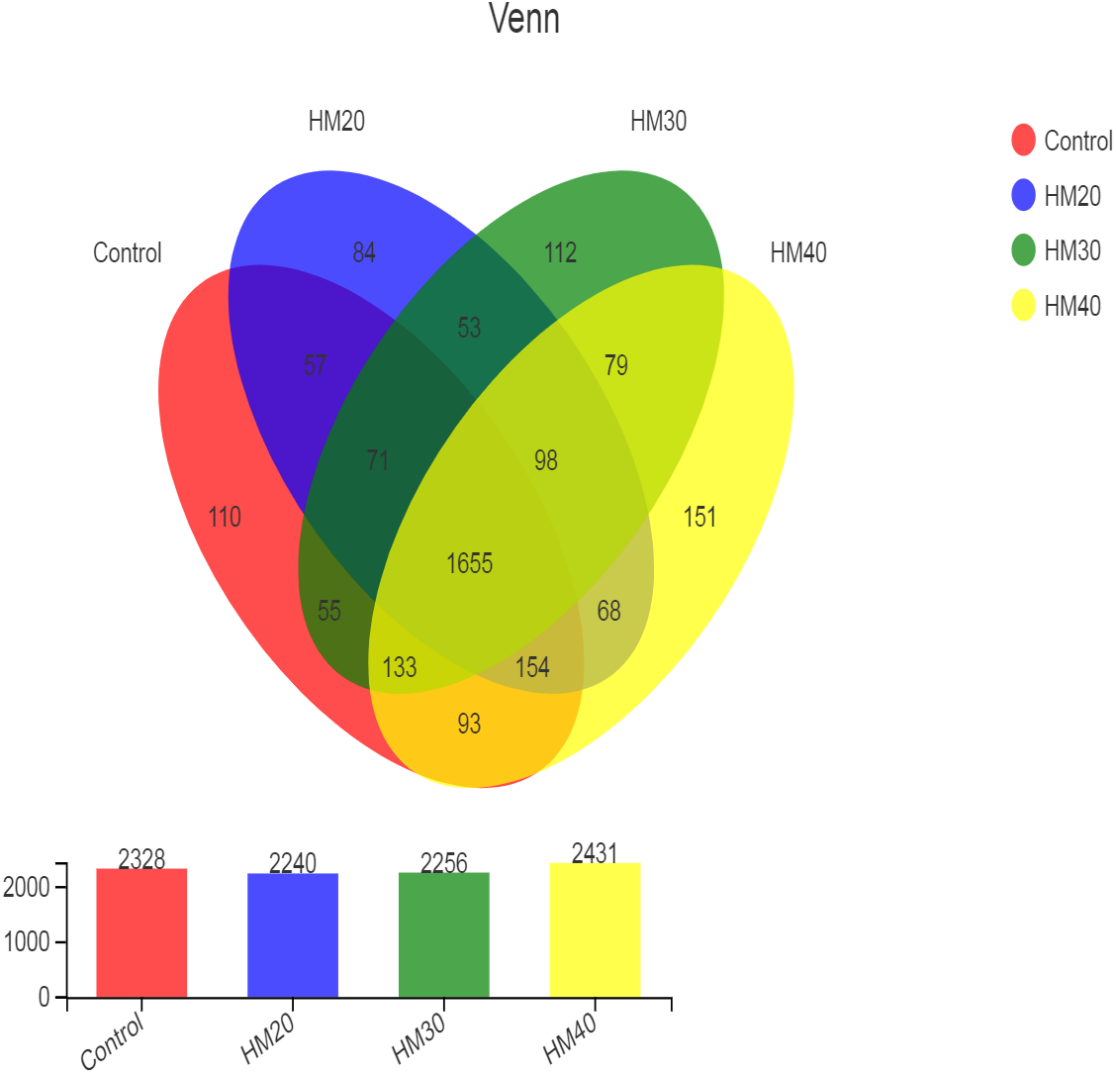
Distribution of OTUs across different treatment groups.

High throughput sequencing of the 16S rRNA gene revealed a total of 2973 OTU in all rumen contents collected from buffaloes. After quality control, these OTUs were classified into 22 phyla, 34 classes, 79 orders, 149 families, 353 genera and 689 species of rumen bacteria. The distribution of shared and unique OTUs for four treatment groups is presented in Fig. 1. The highest numbers of OTU were observed in HM40 as compared with control and other groups. The number of OTU was decreased in HM20 and HM30 but increased in HM40 as compared with the control. A total of 1655 OTU were shared by all groups, whereas the total number of unique OTU was 457 across the four treatment groups. The highest count of unique OTU were found in HM40 (151) followed by HM30 (112), control (110) and HM20 (84) as presented in Fig. 1.

### Alpha diversity indices

Treatment showed no effect (*p* > 0.05) on all alpha diversity parameters analyzed in the present study (Table 5).

### Beta diversity

**Figure 2:**
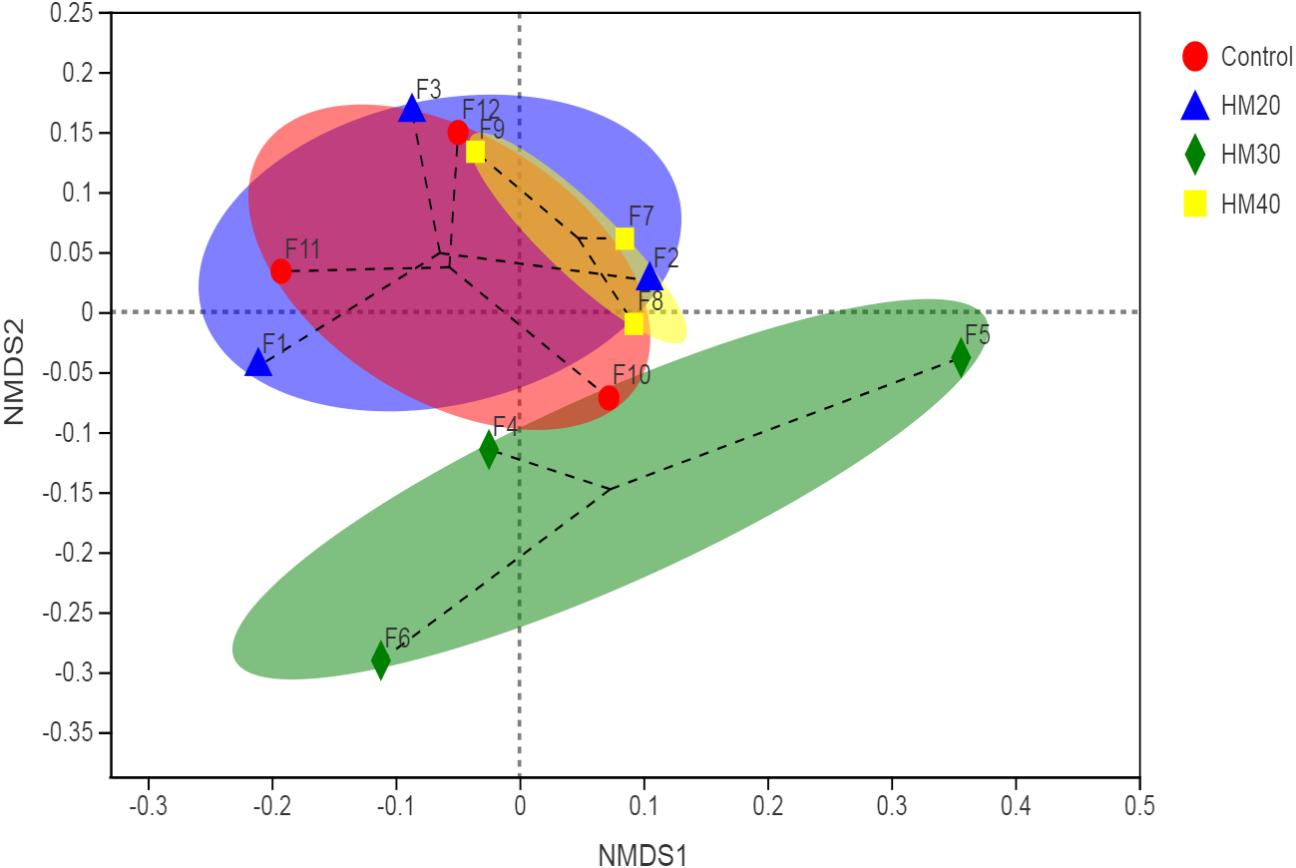
First two dimensions from the (non-metric) multi-dimensional scaling of the Bray-Curtis dissimilarity matrix. Samples were grouped by phytogenic additives. PERMANOVA amongst all groups (*p* = 0.542) using 999 permutations.

The non-metric multidimensional scaling (NDMS) of the Bray-Curtis dissimilarity matrix (first two dimensions) showed the non-significant distance between four treatment groups, as presented in Fig. 2 (*p* = 0.542, from PERMANOVA using 999 permutations).

### The relative abundance of bacterial taxa

**Figure 3:**
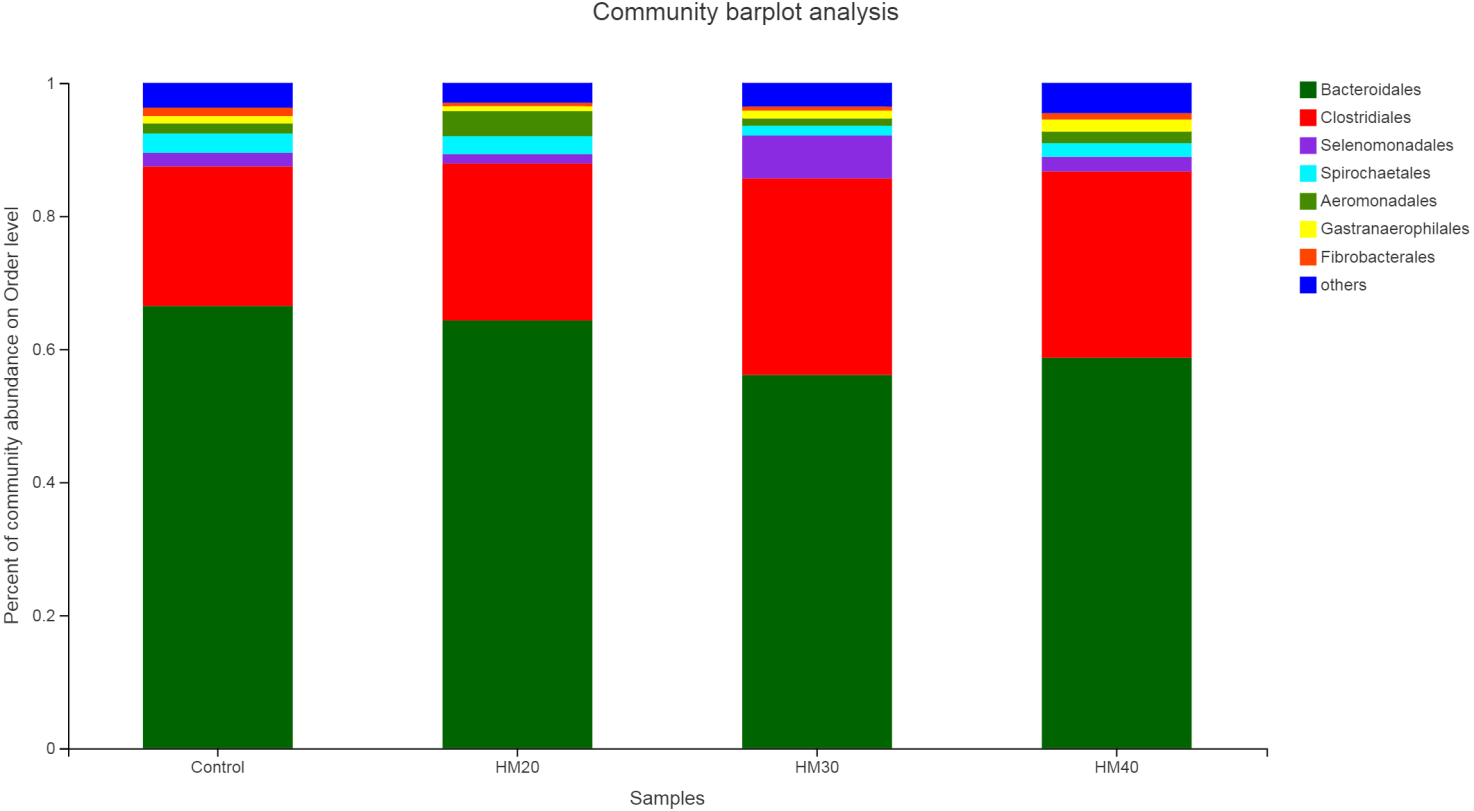
Relative abundance of bacterial phyla across different treatment groups.

The relative abundance of bacterial taxa showed *Bacteroidetes* and *Firmicutes* as dominant phyla representing about 87% of total rumen bacteria observed in buffaloes (Fig. 3, Table S1). Remaining 13% of the bacterial population consisted of *Spirochaetes*, *Protobacteria, Cyanobacteria*, and *Fibrobacteres,* respectively. Relative abundance of *Bacteroidete* was lower in HM20 (64.56%), HM30 (56.15%) and HM40 (58.83%) compared with the control (66.73%) as shown in Table S1. But higher *Firmicutes* community was observed in HM30 (36.19%) as compared with HM20 (25.23%), HM30 (30.48%) and control (23.30%) groups (Fig. 3). The third most abundant phyla was *Proteobacteria* with higher relative abundance in HM20 (4.91%) as compared with HM30 (2.03%) and HM40 (3.35%) compared with control (2.82%). However, *Cyanobacteria* was higher in HM40 (1.81%) as compared with HM20 (0.76%), HM30 (1.17%), HM40 (1.72%) and control group (1.09%). Notably, these differences were not significant (*P* > 0.05).

**Table 5:** Effect of herbal mixture on alpha diversity parameters of rumen bacteria in buffaloes.

**Parameter**	**Control**	**HM20**	**HM30**	**HM40**	**SEM**	***P*value**
**Shannon**	5.76	5.59	5.71	5.89	0.062	0.433
**Simpson**	0.010	0.012	0.012	0.009	0.001	0.425
**Ace**	2071.5	1959.2	2039.1	2178.4	46.274	0.462
**Chao**	2095.7	2002.2	2046.2	2220.6	44.902	0.387
**Shannonevenness**	0.777	0.763	0.775	0.789	0.006	0.562
**Simpsonevenness**	0.060	0.055	0.056	0.065	0.003	0.807

**Notes.**

Values in the same row with different superscripts differ significantly (*P* < 0.05).

(HM20 = herb mixture fed 20 g/d/head, HM30 = mixed herb mixture fed 30 g/d/head, HM40 = herb mixture fed 40 g/d/head, control = without herb mixture).

**Figure 4:**
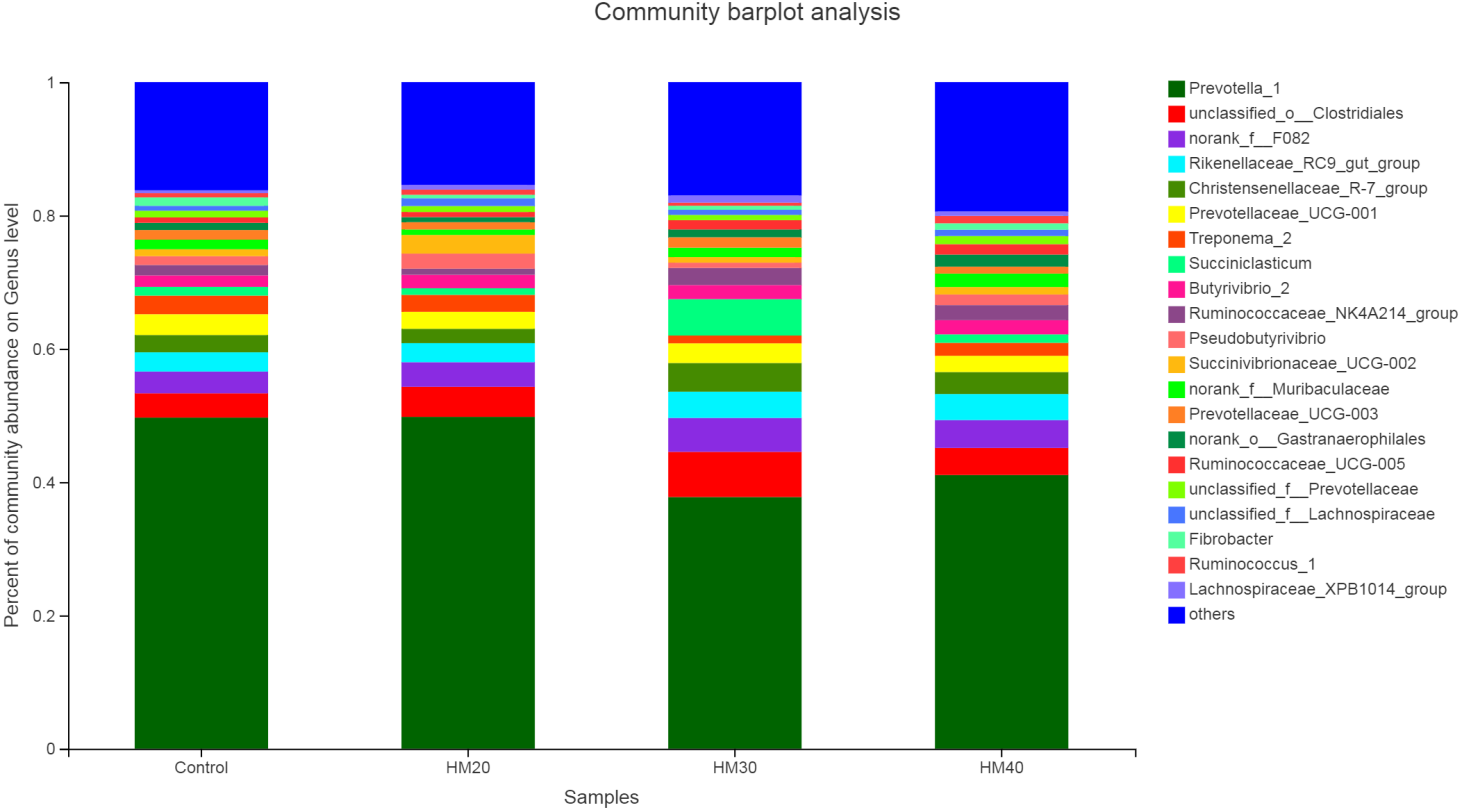
Relative abundance of bacterial genera across treatment groups.

Similar to bacteria phyla, no significant effect of treatment on the relative abundance of bacterial genera was observed (Table S2). Nevertheless, *Prevotella* as a dominant genus (37 to 50% of total bacteriome) was detected in the rumen contents of buffaloes in the present study. Relative abundance of *Prevotella* was higher in HM20 (51.16%) and control group (49.12%) as compared with HM30 (39.93%) and HM40 (40.92%) groups (Fig. 4). The second most abundant genus was *unclassified*-o-*Clostridales,* which showed higher abundance in HM30 (6.20%) as compared with HM20 (4.29%), HM40 (4.08%) and control (3.71%). Moreover, abundance of *Rikenellaceae* also increased in HM30 (3.74%) and HM40 (3.92%) while decreased in HM20 (2.72%) as compared with the control group (3.08%). The highest abundance of *Christensenellaceae R7 group* was observed in HM30 (3.81%) and HM40 (3.39%) as compared with HM20 (2.06%) and control (2.63%). Substantially higher abundance of *Succiniclasticum* was observed in HM30 (4.66%) as compared with HM20 (1.01%), HM40 (1.28%) and control (1.33%). . Interestingly, *Pseudobutyrivibrio* was very low in HM30 (0.84%) but showed higher abundance in HM20 (2.12%) as compared with HM40 (1.59%) and control (1.26%) groups.

### Association of rumen bacteria with rumen fermentation parameters

**Figure 5:**
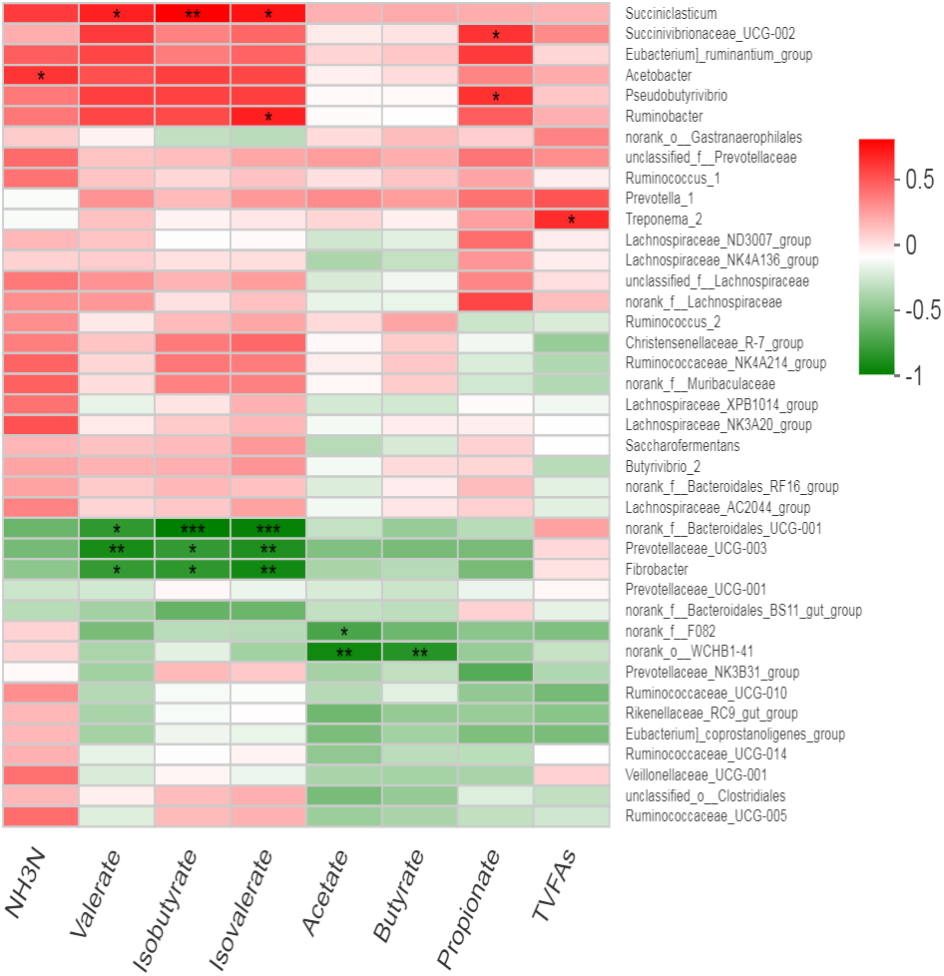
Correlation of bacterial genera with rumen fermentation parameters. In the two-dimensional heat map, change in defined color and its depth indicates the nature and strength of the correlation, respectively. Asterisk sign was used when the r value was greater than 0.1 and the *p* values were less than 0.05 (* 0.01 < *p* ≤ 0.05, ** 0.001 < *p* ≤ 0.01, *** *p* ≤ 0.001).

We observed no effect of treatment on the relative abundance of bacteria; therefore, all samples were used collectively to calculate overall correlation of relative abundance of bacterial genera (having abundance >1%) with rumen fermentation parameters. We observed several significant correlations between bacterial genera and ruminal VFA (Fig. 5, Table S3). *Acetobactor* showed positive correlation with NH_3_-N (*r* = 0.58, *p* < 0.05) while *Fibrobactor* showed negative correlations with valerate (*r* =  − 0.68, *p* < 0.05), isovalerate (*r* =  − 0.79, *p* < 0.01) and isobutyrate (*r* =  − 0.70, *p* < 0.05). Similarly, an uncharacterized genus of *Prevotella* (*Prevotellaceae* _UCG-003) showed negative correlations with valerate (*r* =  − 0.77, *p* < 0.01), isovlerate (*r* =  − 0.75, *p* < 0.01) and isobutyrate (*r* =  − 0.69, *p* < 0.05). However, *Pseudobutyrivibrio* showed positive correlation with propionate concentration (*r* = 0.60, *p* < 0.05). Similarly, *Ruminobactor* showed positive correlation with isovalerate concentration (*r* = 0.65, *p* < 0.05). However, *Succiniclasticum* showed positive correlation with valerate (*r* = 0.65, *p* < 0.05), isovalerate (*r* = 0.69, *p* < 0.05) and isobutyrate (*r* = 0.75, *p* < 0.01). *Succinibrionaceae* _UCG-002 was positively correlated with propionate (*r* = 0.59, *p* < 0.05) and valerate (*r* = 0.57, *p* = 0.05). Moreover, *Treponema* _2 showed positive correlation with total volatile fatty acids (*r* = 0.61, *p* < 0.05). Furthermore, an uncharacterized strain *f__Bacteroidales* _UCG-001 showed strong negative correlations with valerate (*r* =  − 0.70, *p* < 0.05), isovlerate (*r* =  − 0.83, *p* < 0.01) and isobutyrate (*r* =  − 0.85, *p* < 0.01). Similarly, two uncharacterized strains *f__* F082 and *o__WCHB1*-41 showed negative correlation with acetate and butyrate. The *Prevotellaceae* _NK3B31_group was negatively correlated with ruminal propionate (*r* =  − 0.58, *p* = 0.05) concentration.

### Association of rumen bacteria with milk yield and composition

**Figure 6:**
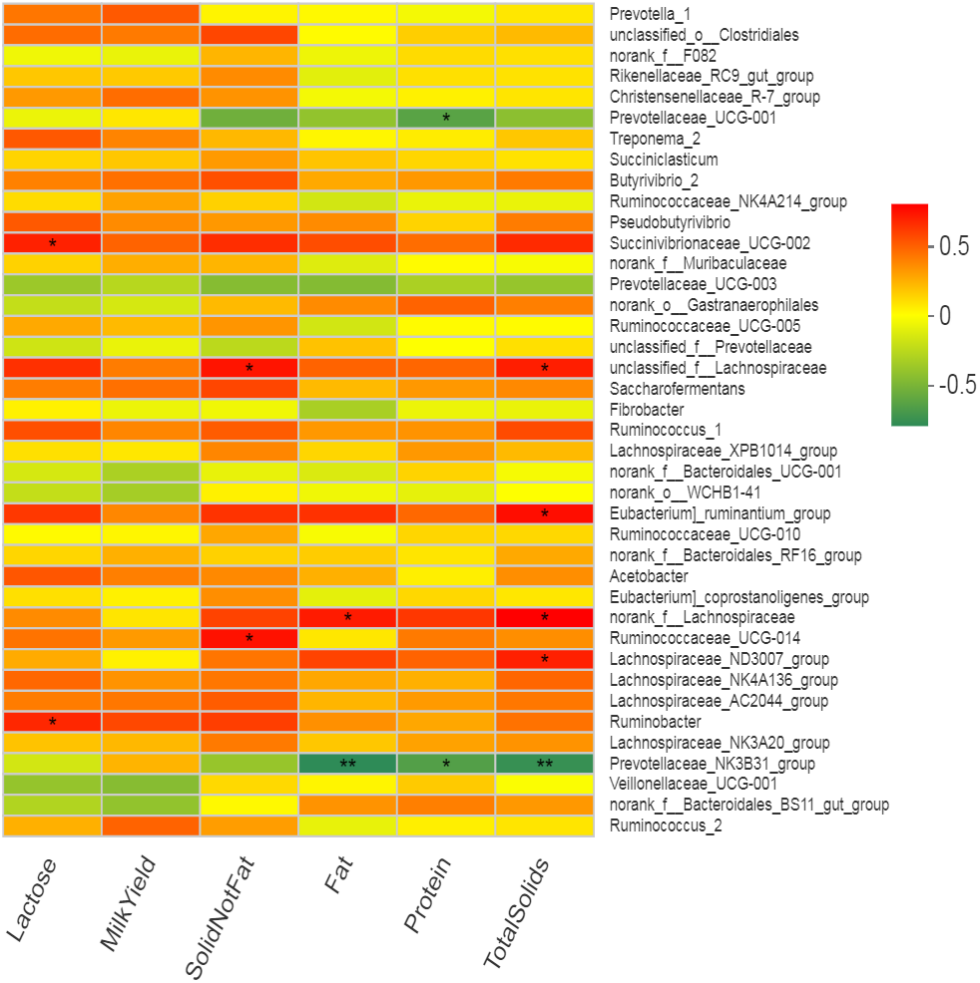
Correlation of bacterial genera with milk yield parameters. In the two-dimensional heat map, change in defined color and its depth indicates the nature and strength of the correlation, respectively. Asterisk sign was used when the r value was greater than 0.1 and the *p* values were less than 0.05 (* 0.01 < *p* ≤ 0.05, ** 0.001 < *p* ≤ 0.01, *** *p* ≤ 0.001).

Many bacterial genera showed several correlations with different milk composition traits but no significant correlation of any bacterial genus with milk yield was observed. Five bacterial genera (including *Eubacterium_ruminantium* _group, norank_f*__Lachnospiraceae, Succinivibrionaceae_* UCG-002, unclassified_f__*Lachnospiraceae* and *Lachnospiraceae* _ND3007_group) showed positive correlation (*r* = 0.57 to 0.69, *p* < 0.05) while only one genus (*Prevotellaceae* _NK3B31_group) showed negative correlation (*r* =  − 0.71, *p* < 0.05) with total solids in milk (Fig. 6, Table S4). Two bacterial genera (*Prevotellaceae_* UCG-001 and *Prevotellaceae_* NK3B31_group) showed highly significant (*p* < 0.01) negative correlation (*r* =  − 0.59 and −0.62, respectively) with milk protein (%). The *Lachnospiraceae* showed positive correlation (*r* = 0.62, *p* < 0.05) while *Prevotellaceae_NK3B31_group* showed negative correlation (*r* =  − 0.74, *p* < 0.01) with milk fat (%). Two bacterial genera (*Ruminococcaceae_* UCG-014 and *unclassified_f__Lachnospiraceae)* showed significantly (*p* < 0.05) positive correlation (*r* = 0.64) with solid not fat (SNF) content of milk. Two bacterial genera (*Succinivibrionaceae_* UCG-002 and *Ruminobacter)* showed significant (*p* = 0.05) positive correlation (*r* = 0.60 and 0.58 respectively) with milk lactose content. Only one bacterial strain *Ruminobacter* was positive correlated (*r* = 0.59, *p* < 0.05) with protein yield. However, three bacterial strains (*Prevotella_1, Succinivibrionaceae_* UCG-002 and *Ruminobacter)* showed significant positive correlations (*r* = 0.60, 0.59, 0.59, respectively) with milk fat yield.

### Association of rumen bacteria with milk fatty acid contents

**Figure 7:**
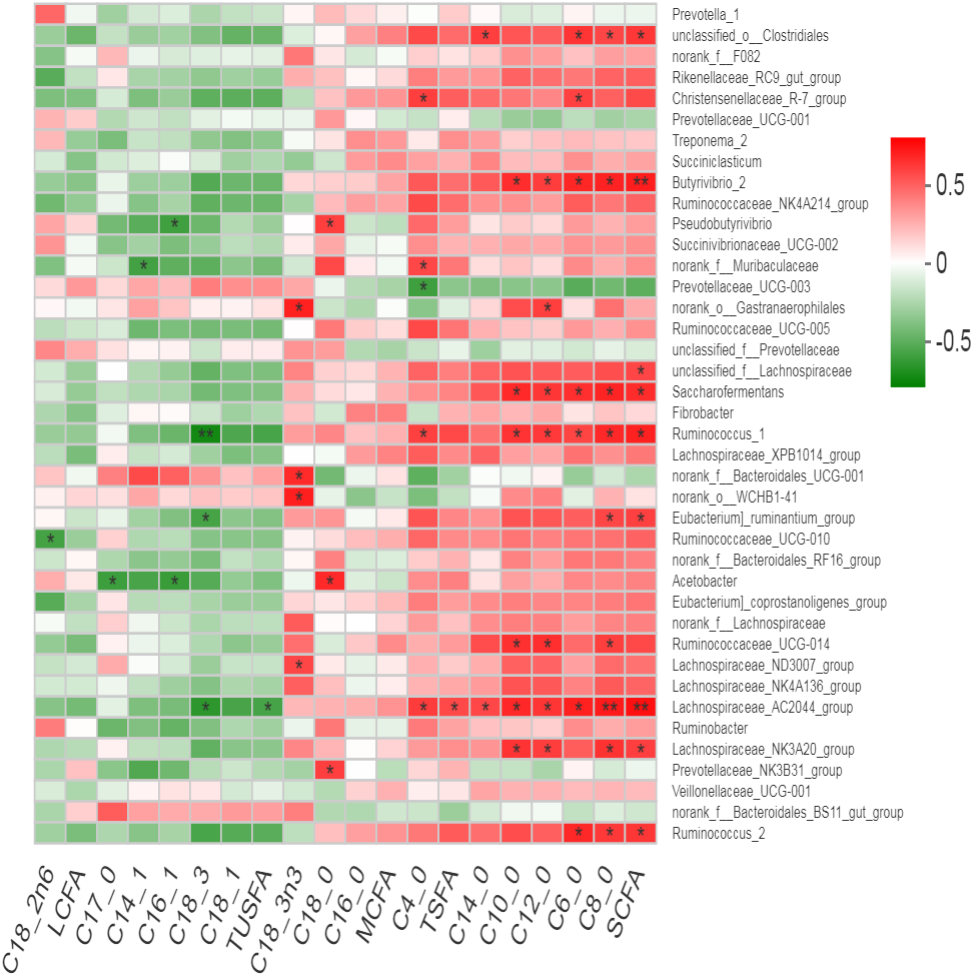
Correlation of bacterial genera with milk fatty acid contents.

Many bacterial genera showed positive correlation with the milk fatty acid contents (Fig. 7). An un-characterized genus of rumen bacteria “*unclassified-o-Clostrdiales* showed positive correlation with total SCFA along with C4:0, C8:0 and C14:0. *Christensenellaceae R7 group* showed positive correlation with C4:0 and C6:0 while *norank_o__Gastranaerophilales* showed positive correlation with C12:0 only. *Butyrivibrio, Saccharofermntas and Ruminococcus1* positively correlated with total SCFA along with C6:0, C8:0, C10:0 and C12:0 contents. *Ruminococcaceae_* UCG-014 showed positive correlation with C8:0, C10:0 and C12:0 contents while *Ruminococcus2* positively correlated with total SCFA, C6:0 and C8:0 contents of milk. *Lachnospiraceae_AC2044_group* exhibited positive correlation with total SCFA, C4:0, C6:0, C8:0, C10:0, C12:0, C14:0 and TSFA contents but showed negative correlation with TUSFA and C18:3. However, *Lachnospiraceae _NK3A20_group* positively correlated with total SCFA, C8:0, C10:0 and C12:0 contents. As a major saturated fatty acid, C18:0 (stearic acid) showed positive correlation with three bacterial genera including *Prevotellaceae_NK3B31_group, Acetobacter* and *Pseudobutyrivibrio.* In contrast*,* C18:3n3 positively correlated with *Lachnospiraceae_ND3007_group, norank_o__WCHB1*-41, *f__Bacteroidales* _UCG-001 and *norank_o__Gastranaerophilales. Eubacterium_ruminatum_group* showed positive correlation with total SCFA including C8:0 while negative correlation with C18:3 content. *Acetobacter* showed negative correlation with C17:0 and C16:1. *Ruminococcaceae_* UCG-014 showed negative correlation with C18:2n6.

## Discussion

### DMI, milk yield and composition

The non-significant effect of herb mixture on DMI observed in this study, has also been reported earlier in dairy cattle (Benchaar, 2016; Benchaar et al., 2012; Oh et al., 2018) and buffaloes (De Paula et al., 2016). Dietary supplementation of garlic and peppermint also revealed no effect on DMI and nutrient digestibility in buffaloes (Verma et al., 2012). Due to the established association of rumen fermentation parameters with milk yield (Seymour, Campbell & Johnson, 2005), no subsequent changes in milk yield traits were observed in the present study due to absence of treatment effects on rumen fermentation. Studies using a blend of different phytochemicals like cinnamaldehyde, eugenol, and capsicum have also shown non-significant effects on milk yield in dairy cattle (Oh et al., 2013). Similarly, no effect of eugenol was observed on milk yield in cows (Oh et al., 2013). Moreover, the combination of eugenol and cinnamaldehyde also showed no significant change in the productive performance of dairy cattle (Tager & Krause, 2011; Tekippe et al., 2013).

A tendency of increase in milk fat (%) was observed in buffaloes supplemented with herbs mixture as compared with the control group. Milk fat content is related to acetate and butyrate concentrations which are precursors of a diverse range of compounds in the body especially fatty acids and total cholesterol (Pennington, 1952). Moreover, their concentrations are directly related to fermentation kinetics in the rumen. Similar findings have been reported earlier regarding the effects of supplementation of peppermint in dairy cows showing a non-significant change in DMI, milk yield and composition except milk fat (Hosoda et al., 2005).

Our study revealed C16:0 and C18:1 as the major fatty acids followed by C18:0 and C14:0 which is in agreement with earlier studies in dairy cattle (Heck et al., 2012; Pegolo et al., 2016). Contents of SFA (62–64%) and UFA (36–38%) observed in our study are similar to earlier reports in cattle and buffaloes (Abdullah et al., 2019; Ebeid, Gawad & Mahmoud, 2015). Dietary polyphenolic compounds have shown to manipulate microbial biohydrogenation in the rumen which can lead to increase desirable fatty acid contents of milk (Butler et al., 2011; Collomb et al., 2008; Ellis et al., 2006). Significant increase observed in linoleic (up to12%) and linolenic acid (7.5 to 13%) in HM20 in present study is in agreement with earlier studies that reported up to 30% increase in these milk fatty acids in response to feeding of polyphenolic rich forage (condensed tannins) in sheep (Cabiddu et al., 2009; Roy et al., 2002). This increase in UFA contents coupled with decrease in major SFA (Stearic acid; C18:0) up to 10% mediated by herb mixture reflects their dual positive effects regarding human health point of view. These findings are mainly attributed to the ability of polyphenolic compounds to decrease biohydrogenation of dietary fatty acids in the rumen by selective modulation of specific microbes leading to proportional increase in UFA (Cabiddu et al., 2010; Vasta et al., 2008). It was evident by an increase in the relative abundance of *Butyrivibrio* species in response to treatment, which is reported to have a positive correlation with linolenic acid and n-3 fatty acids in milk (Bainbridge et al., 2016). Moreover, other bacteria taxa also contributed to higher contents of UFA in HM20 owing to their higher abundance and potential association with milk fat content as mentioned above.

Since we did not determine fatty acid contents of rumen microflora, we are unable to directly associate bacterial abundance with the fatty acid profile in milk. This limitation should be accounted for in future studies. Our study demonstrated that herb mixture can alter rumen bacterial populations and manipulate the rumen biohydrogenation resulting in an increase in milk fat and PUFA contents but studies on a larger cohort are required to acquire statistical significance and corroborate these findings.

### Rumen bacterial diversity and fermentation parameters

We attempted in evaluating sustainable and long-term effects of phytochemicals on rumen microbiota, so rumen sampling was carried out once before feeding after 10 weeks of treatment. Moreover, we tried to minimize animal-to-animal variation on the host side by selecting animals with the same parity, stage of lactation, and body weight, which was evident by similar rumen fermentation and milk yield parameters observed in all treatment groups. So, we assumed that variations observed in diversity and relative abundance of rumen bacteria are mainly attributed to the effect of herb mixture. Results of relative abundance of major bacterial orders observed in our study are in agreement with earlier studies reporting *Bacteroidetales* and *Clostridiales* as dominant order that represent more than 87% of total bacteriome in dairy cattle and buffaloes (Oh et al., 2013; Zhan et al., 2017).

Despite of the substantial variation observed across treatments groups as compared to the control, effect of treatment on the relative abundance of rumen bacteria was non-significant, which requires further studies on larger cohort to corroborate these findings. For example, we observed about 9% decrease in *Prevotella* in HM40 (49.12 vs 40.92) as compared to the control which is even higher than significant decrease observed in this bacterial genus in high producing cows (39% vs. 48) as compared to low producers as reported previously (Mu et al., 2018). The *Prevotella* is a major bacterial genus of rumen bacteria with well-defined role in dietary protein degradation in particular and feed digestibility in general. The abundance of *Prevotella* as a dominant genus in buffalo rumen has also been widely reported in earlier studies (Li et al., 2009). Moreover, the decrease in *Prevotella* with the medium (HM30) and high levels (HM40) was associated with lower NH_3_-N concentration (though non-significant) in both groups of buffaloes (Bi et al., 2018). Interestingly, substantially higher abundance of *Succiniclasticum* was observed in HM30 as compared with other groups which seem to be associated with decreased abundance of *Prevotella*. A strong negative correlation of *Prevotella* has been observed with *Succiniclasticum* and *Ruminococcus* in buffalo rumen (Iqbal et al., 2018). Major polyphenolic compounds like flavonoids and saponins are degraded through deglycosylation by gut microbes. This fact might have contributed to the overall non-significant effects on diversity and relative abundance of bacteria observed in the present study.

Our study revealed no effect of treatment on rumen fermentation parameters which is in agreement with earlier study regarding dietary inclusion of garlic and peppermint in buffaloes (Verma et al., 2012). These findings may be attributed to the fact that rumen microbes adapt to different phytochemicals over time and restore their fermentation activities, but the effectiveness of the adaptation depends on the robustness and diversity of the microbiome, length of exposure, and the effective dose of inhibitor (Cobellis et al., 2016). An *in vitro* study reported no effect of three plant extracts (garlic, cinnamon, and aniseed) after 6 days of supplementation, although they significantly altered molar proportions of acetate, propionate, and butyrate prior this period (Cardozo et al., 2004). This is the main reason for our observation of non-significant effects on rumen fermentation parameters in the present study, in addition to lower sample size. However, highly variable results regarding shifts in rumen fermentation patterns in response to treatment with herbs have been reported. Besides positive and/or negative changes, even no significant effects of phytochemicals (plant extracts or essential oils) on rumen fermentation end products have been observed. These divergent findings may be partially explained by variable experimental conditions of studies including the type of diets, plant species, dose and type of active phytochemicals, pH of rumen fluid and host animal (Christaki et al., 2012; Tajodini et al., 2014). It has been suggested that using a combination of different plant compounds (with different potential activities) would lead to the synthesis of new metabolites during rumen fermentation, with quite different bioactivities (Newbold et al., 2004; Spanghero et al., 2009). Although this approach makes it difficult to screen individual causative effects of phytochemicals still it is an exciting area to explore and develop phytogenic interventions for modulation of rumen microbiome to improve the performance of ruminants in terms of milk yield and composition particularly fatty acid profile. It is particularly relevant and imperative to look for natural feed additives to replace antibiotic growth promoters in animal feeding.

### Association of rumen bacteria with rumen fermentation and milk yield parameters

Rumen bacteria are the most abundant and diverse group of microbes that constitute more than 95% of the total rumen microbiome (Flint et al., 2008). The major role of rumen bacteria is the degradation of plant polysaccharides (Flint et al., 2008) to produce VFA as the main source of energy for animals (Mizrahi, 2012). Production of VFA in the rumen is directly associated with rumen bacteriome and subsequent epithelial absorption by the animal (Brockman, 2005). That is why bacterial activities in the rumen directly affect the milk yield and composition along with other physiological characteristics in ruminants (Hurtaud, Rulquin & Verite, 1993). In the present study, Spearman’s correlation analysis revealed the relationship of various bacterial genera with rumen fermentation parameters exhibiting many positive and negative associations. Overall, 16 positive and four negative correlations of bacterial genera with milk yield parameters were observed. However, we observed 12 positive and 14 negative correlations of rumen bacteria with rumen VFA in present study. Observation of non-significant correlation with milk yield but many positive correlations with milk components is in agreement with earlier findings that milk fat and protein percentages are more likely to be correlated with rumen bacterial communities as compared with milk production (Zhu & Noel, 2016). Most of the correlations were exhibited by well-known cellulolytic, amylolytic and proteolytic bacterial genera with an established role in fiber, starch, and protein breakdown, respectively (Jami, White & Mizrahi, 2014; Jiang et al., 2017; Zou et al., 2019). Previously reported correlation of *Butyrivibrio* with milk fat yield, milk total solids and total milk yield in buffalo was not observed in this study (Zou et al., 2019). However, a negative correlation of *Prevotella* with milk fat (%) observed in the present study was in agreement with earlier reports on dairy cows (Jami, White & Mizrahi, 2014; Jiang et al., 2017).

Earlier studies reported that polyphenolic rich forage increased the *α*-linoleic acid content of milk in sheep (Cabiddu et al., 2009; Roy et al., 2002). The decrease in stearic acid (C18:0) together with the increase in n-6 fatty acid contents of milk, is in agreement with earlier studies which have reported similar findings with supplementation of tannins in dairy sheep (Buccioni et al., 2015). Based on the ratio of C14:1 to C14:0 (a proxy of desaturation), it has been suggested that polyphenols (tannins) can enhance the activity of stearoyl Co-A desaturase enzyme (SCD), which mediates the conversion of stearic acid to oleic acid and vaccenic acid to conjugated linolenic acid (CLA). In particular, SCD has shown to contribute almost 50% of oleic acid and cis-9, trans-11 CLA secreted in sheep milk (Frutos et al., 2014). This implies that polyphenols can increase milk unsaturated fatty acids especially n-3 and n-6 fatty acids not only by mediating rumen biohydrogenation but also enhancing SCD activity (Buccioni et al., 2015; Mele et al., 2007; Vasta et al., 2009b).

### Association of rumen bacteria with milk fatty acid contents

The present study revealed overall 52 positive and 10 negative correlations of rumen bacteria with milk fatty acid contents. The negative correlation of *Ruminococcaceae_* UCG-014 with C18:2n6 is in agreement with earlier studies reporting negative association of *Ruminococcaceae* family with PUFA in dairy goat (Cremonesi et al., 2018). Milk fat is considered as an important economic factor in the dairy industry. The ruminal microbes contribute to milk fat thesis through two main processes in the rumen; (1) Digestion of soluble and insoluble carbohydrates to produce VFA such as acetic and butyric, which are oxidized to acetyl CoA (via TCA cycle) and subsequently serve as precursors of milk fat synthesis especially SCFA. (2) Microbes convert PUFA into saturated fatty acids through biohydrogenation (BH) process (Jenkins et al., 2008). Available information about the effect of phytogenic herbs on milk fatty acid content is limited than on rumen BH (Toral et al., 2018). However, the plant secondary compounds have shown favorable effects on modulation of rumen BH (Vasta et al., 2009a). Microbial BH process mediated by bacterial genera such as *Butyrivibrio* and *Pseudobutyrivibrio* may accumulate a wide range of intermediates, including rumenic acid, which is reduced to vaccinic acid and finally to C18:0 (Palmquist et al., 2005). This is in agreement with our findings regarding positive correlation of *Pseudobutyrivibrio* with C18:0 which is one of the most abundant SFA in milk. In the present study, many positive correlations of short and medium chain fatty acids with cellulolytic bacteria like *Ruminococcus* species, *Butyrivibrio, Eubacterium_ruminatum_group* and *unclassified-o-Clostrdiales* were observed. This is attributed to the fact that cellulolytic bacteria produce acetate as major end product from fiber degradation, which is subsequently used in the *de novo* milk fatty acid synthesis. Furthermore, the *Lachnospiraceae* groups are a member in the orde*r Clostridiales* that ferment polysaccharides to SCFAs (butyrate, acetate) in the rumen (Boutard et al., 2014). As mentioned before, both of acetate and butyrate act as precursors of milk fat synthesis especially SCFA in milk through TCA cycle. This fact supports the positive correlation of *Lachnospiraceae* (especially *Lachnospiraceae_AC2044_group*) with SCFA and SFA as well as a negative correlation of *Lachnospiraceae_AC2044_group*, *Ruminococcus* and *Eubacterium_ruminatum_group* with C18:3. Positive association of milk C18:3n3 content with *Lachnospiraceae_ND3007_group, norank_o__WCHB1*-41, *f__Bacteroidales* _UCG-001 and *norank_o__Gastranaerophilales* is potentially useful to increase unsaturated fatty acid in milk through dietary interventions.

Therefore, our findings support the earlier findings (Bernard, Leroux & Chilliard, 2008; Shingfield, Bonnet & Scollan, 2013), that rumen microbial fermentation regulates the fat composition of milk by providing precursors (VFA; acetate and butyrate) for de novo FA synthesis in the mammary gland through enhancing the outflow of beneficial FA from the rumen metabolism. Moreover, this metabolic pathway usually yields saturated fatty acid of up to C16, which can subsequently serve as substrates for desaturases and, in some tissues, elongases (Bernard, Leroux & Chilliard, 2008). Our findings envisaged that milk fatty acid composition could be favorably modulated through modulation of rumen microbes by using herbal mixtures.

Overall, our study provides insights into the modulation of rumen bacteria by phytochemicals to improve rumen fermentation parameters and milk yield in buffaloes. Desirable effects regarding an increase in milk fat (%) and PUFA contents while decrease in milk saturated fatty acids are advantageous in terms of economics and the human health point of view. Overall, these findings will also contribute to our understanding of the effects of herbs on rumen bacteria and their respective association with rumen fermentation, milk fatty acid contents and milk yield traits. However, future studies are required involving larger cohorts to elucidate correlation network involving rumen bacteria and their fatty acid contents, VFA and milk yield to provide insights on the modulation of the metabolic network by phytochemicals and prediction of production traits from bacteriome structure.

## Conclusions

Dietary supplementation of herbal mixture had no effects on milk performance, ruminal fermentation, and bacterial diversity in water buffaloes. A tendency to increase in milk fat (%) is advantageous particularly in the absence of increase in fat yield. A significant increase in PUFA contents in HM20 revealed that 20g/day is an appropriate dose of HM for dietary supplementation in water buffalo. Additionally, supplementation of HM promoted the rumen bacteria (*Succinivibrionaceae, Butyrivibrio, Pseudobutyrivibrio*, and *Lachnospiraceae*) that are positively associated with milk yield, fat yield and milk fatty acid contents. Positive association of milk C18:3n3 content with *Lachnospiraceae_ND3007_group, norank_o__WCHB1*-41, *f__Bacteroidales* _UCG-001 and *norank_o__Gastranaerophilales* reveals their potential utility for increasing PUFA content of buffalo milk. Nevertheless, further studies on larger cohorts are required to corroborate these findings.

##  Supplemental Information

10.7717/peerj.11241/supp-1 Supplemental Information 1 Supplementary Tables

10.7717/peerj.11241/supp-2 Supplemental Information 2 Arrive Guidelines Author Checklist

10.7717/peerj.11241/supp-3 Supplemental Information 3 Raw data of Rumen fermentation parameters

10.7717/peerj.11241/supp-4 Supplemental Information 4 Raw data for Fatty Acid content of Milk

10.7717/peerj.11241/supp-5 Supplemental Information 5 Raw data of Milk yield and composition

## Additional Information and Declarations

### Data Availability

The following information was supplied regarding data availability: Raw data are available in the Supplemental Files and the sequences are available at BioProject PRJNA564158.
