# Supplementary material for: Consequences of herbal mixture supplementation on milk performance, ruminal fermentation, and bacterial diversity in water buffaloes
Source: PeerJ. 2021 May 14;9:e11241. doi: 10.7717/peerj.11241 (PMC8127954; doi:10.7717/peerj.11241)
Supplement: Supplemental Information 1 [file peerj-09-11241-s001.docx]

**Table S-1 Relative abundance of major bacterial orders observed in different treatment groups of buffaloes**

| **Species name** | **Control (%)** | **HM20 (%)** | **HM30 (%)** | **HM40 (%)** | **P-value** | **Corrected P-value** |
| --- | --- | --- | --- | --- | --- | --- |
| Bacteroidetes | 66.73 | 64.56 | 56.15 | 58.83 | 0.62 | 0.97 |
| Firmicutes | 23.30 | 25.23 | 36.19 | 30.48 | 0.32 | 0.97 |
| Proteobacteria | 2.82 | 4.91 | 2.03 | 3.35 | 0.83 | 0.99 |
| Spirochaetes | 2.94 | 2.75 | 1.50 | 2.07 | 0.35 | 0.97 |
| Cyanobacteria | 1.09 | 0.76 | 1.17 | 1.81 | 0.58 | 0.97 |
| Fibrobacteres | 1.27 | 0.52 | 0.62 | 0.96 | 0.89 | 0.99 |
| others | 1.85 | 1.26 | 2.35 | 2.50 | 0.36 | 0.97 |

**Table S-2 Relative abundance of major bacterial genera observed in in different treatment groups of buffaloes**

| **Species name** | **Control (%)** | **HM20 (%)** | **HM30 (%)** | **HM40 (%)** | **P-Value** | **Corrected P-value** |
| --- | --- | --- | --- | --- | --- | --- |
| Prevotella_1 | 49.12 | 51.16 | 39.93 | 40.92 | 0.46 | 0.83 |
| unclassified_o__Clostridiales | 3.71 | 4.29 | 6.20 | 4.08 | 0.76 | 0.88 |
| norank_f__F082 | 3.42 | 3.61 | 5.15 | 4.20 | 0.63 | 0.87 |
| Rikenellaceae_RC9_gut_group | 3.08 | 2.72 | 3.74 | 3.92 | 0.79 | 0.89 |
| Christensenellaceae_R-7_group | 2.63 | 2.06 | 3.81 | 3.39 | 0.67 | 0.87 |
| Prevotellaceae_UCG-001 | 3.11 | 2.60 | 3.24 | 2.44 | 0.58 | 0.83 |
| Treponema_2 | 2.77 | 2.52 | 1.29 | 1.89 | 0.67 | 0.87 |
| Succiniclasticum | 1.33 | 1.01 | 4.66 | 1.28 | 0.56 | 0.83 |
| Butyrivibrio_2 | 1.69 | 1.94 | 1.97 | 2.18 | 0.92 | 0.97 |
| Ruminococcaceae_NK4A214_group | 1.61 | 0.89 | 2.23 | 2.35 | 0.29 | 0.83 |
| Pseudobutyrivibrio | 1.26 | 2.12 | 0.84 | 1.59 | 0.46 | 0.83 |
| Succinivibrionaceae_UCG-002 | 0.94 | 2.61 | 0.91 | 1.11 | 0.35 | 0.83 |
| norank_f__Muribaculaceae | 1.48 | 0.78 | 1.26 | 2.04 | 0.35 | 0.83 |
| Prevotellaceae_UCG-003 | 1.49 | 1.16 | 1.76 | 1.02 | 0.62 | 0.87 |
| norank_o__Gastranaerophilales | 1.11 | 0.78 | 1.24 | 1.75 | 0.58 | 0.83 |
| Ruminococcaceae_UCG-005 | 0.84 | 0.74 | 1.24 | 1.55 | 0.50 | 0.83 |
| unclassified_f__Prevotellaceae | 0.98 | 0.96 | 0.85 | 1.23 | 0.68 | 0.87 |
| unclassified_f__Lachnospiraceae | 0.73 | 1.13 | 0.77 | 0.95 | 0.44 | 0.83 |
| Fibrobacter | 1.32 | 0.53 | 0.70 | 0.92 | 0.89 | 0.95 |

**Table S-3Correlation coefficients (Spearman) between rumen bacterial genera and rumen fermentation parameters**

| **Bacterial Genus** | **Acetate** | **Propionate** | **Isobutyrate** | **Butyrate** | **Isovalerate** | **Valerate** | **TVFAs** | **NH_3_-N** |
| --- | --- | --- | --- | --- | --- | --- | --- | --- |
| norank_f__F082 | -0.61 |  |  | -0.5* |  |  |  |  |
| Treponema_2 |  |  |  |  |  |  | 0.61 |  |
| Succiniclasticum |  |  | 0.75 |  | 0.69 | 0.65 |  | 0.57 |
| Succinivibrionaceae_UCG-002 |  | 0.59 |  |  |  | 0.57* |  |  |
| Prevotellaceae_UCG-003 |  |  | -0.69 |  | -0.75 | -0.77 |  |  |
| Pseudobutyrivibrio |  | 0.60 |  |  |  |  |  |  |
| Fibrobacter |  |  | -0.7 |  | -0.79 | -0.68 |  |  |
| norank_f__Bacteroidales_UCG-001 |  |  | -0.85 |  | -0.83 | -0.7 |  |  |
| norank_o__WCHB1-41 | -0.79 |  |  | -0.73 |  |  |  |  |
| Eubacterium_ruminantium_group | 0.09 |  |  | 0.13 |  |  |  |  |
| Acetobacter |  |  |  |  |  |  |  | 0.58 |
| Ruminobacter |  |  |  |  | 0.65 |  |  |  |
| Prevotellaceae_NK3B31_group |  | -0.58 |  |  |  |  |  |  |

Only significant (P < 0.05 and *P=0.05) correlations are presented.

**Table S-4 Correlation coefficients (Spearman) between rumen bacterial genera and milk yield parameters**

| **Bacterial Genus** | **Protein (%)** | **Fat (%)** | **Total Solids** | **Solid Not Fat** | **Lactose** | **Protein Yield** | **Fat Yield** |
| --- | --- | --- | --- | --- | --- | --- | --- |
| Prevotella_1 |  |  |  |  |  |  | 0.60 |
| Prevotellaceae_UCG-001 | -0.59 |  |  |  |  |  |  |
| Succinivibrionaceae_UCG-002 |  |  | 0.57 |  | 0.60 |  | 0.59 |
| unclassified_f__Lachnospiraceae |  |  | 0.61 | 0.64 | 0.56 |  |  |
| Eubacterium_ruminantium_group |  |  | 0.65 |  |  |  |  |
| norank_f__Lachnospiraceae |  | 0.62 | 0.69 |  |  |  |  |
| Ruminococcaceae_UCG-014 |  |  |  | 0.64 |  |  |  |
| Lachnospiraceae_ND3007_group |  |  | 0.60 |  |  |  |  |
| Ruminobacter |  |  |  |  | 0.58 | 0.59 | 0.59 |
| Prevotellaceae_NK3B31_group | -0.62 | -0.74 | -0.71 |  |  |  |  |

Only significant (P < 0.05) correlations are presented.
